# Enhancement of Antibacterial Activity of Carbon Dots via Lysozyme Coupling

**DOI:** 10.1021/acsabm.5c02068

**Published:** 2026-01-13

**Authors:** Tianxiao Wang, Henry Opoku, Menghong Li, Maria Hedberg, Jia Wang, Wen Kou

**Affiliations:** † School of Chemical Engineering, 12399 Dalian University of Technology, No. 2 Linggong Road, Dalian 116024, China; ‡ Department of Odontology, Umeå University, Umeå 90737, Sweden; § The Organic Photonics and Electronics Group, Department of Physics, Umeå University, Umeå SE-90187, Sweden; ∥ Key Laboratory of Shaanxi Province for Craniofacial Precision Medicine Research, Department of Orthodontics, College of Stomatology, Xi’an Jiaotong University, No.98 Xiwu Road, Xi’an 710004, China; ⊥ Wallenberg Initiative Materials Science for Sustainability, Department of Physics, Umeå University, Umeå SE-90187, Sweden

**Keywords:** carbon dots, lysozyme, biocompatibility, antibacterial, dental caries

## Abstract

To develop a safe, efficient, water-soluble, and targeted antibacterial substance for medical applications, we synthesized carbon dots using citric acid and urea as precursors by a solvothermal method. We then coupled the carbon dots and lysozyme by using a simple 1-ethyl-3-(3′-dimethylaminopropyl) carbodiimide-*N*–hydroxysuccinimide (EDC-NHS) coupling method. After coupling, the carbon dots exhibited improved water dispersibility with particle sizes ranging from 12 to 20 nm. Notably, the highest carbon dot concentration associated with cytotoxicity increased from 2.5 to 5 mg/mL when coupled with lysozyme, implying that coupling could enhance the biocompatibility of carbon nanodots. Furthermore, coupled carbon dots extended the effective inhibition time against *Streptococcus mutans* from 12 to 36 h, compared to carbon dots alone. The improved biocompatibility and prolonged effective antibacterial duration highlight the potential of lysozyme-coupled carbon dots as a safe, efficient, and water-soluble antibacterial agent for a variety of oral healthcare and medical applications.

## Graphical abstract

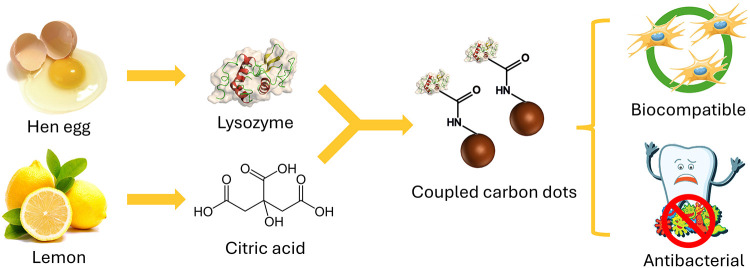

## 1 Introduction

Bacterial infections in the oral cavity, including dental caries, periodontitis, and peri-implantitis, remain major concerns in clinical dentistry. These conditions are largely driven by colonization and biofilm formation of pathogenic microorganisms. Although conventional antibacterial agents have been widely applied to manage these infections, they suffer from important limitations. Chlorhexidine, one of the most frequently prescribed antiseptics, has been reported to cause cytotoxic effects on gingival and periodontal ligament cells,^1^ and its long-term use can lead to tooth staining,^2^ hypogeusia,^3^ and calculus formation.^4^ Similarly, alcohol-based formulations may result in burning sensations, mucosal irritation,^5^ and the production of carcinogenic metabolites such as acetaldehyde.^6^ These drawbacks underscore the urgent need for safer, biocompatible, and water-soluble antibacterial materials with targeted activities for oral healthcare.

Nanomaterials have attracted increasing attention as innovative solutions to this challenge. Among them, carbon dots (CDs) represent a promising class due to their facile synthesis, tunable surface functionality, photoluminescence, and favorable biocompatibility.^7^ CDs have been explored for a wide range of biomedical purposes, including antibacterial activity, fluorescence imaging, and photodynamic therapy.^8^ Importantly, their surface chemistry can be readily modified to enhance antibacterial efficacy and to adapt them for specific clinical uses.

Lysozyme (LZM), a naturally occurring antibacterial enzyme abundant in saliva, tears, and other human secretions, is a strong candidate for functionalization.^9^ LZM displays excellent biocompatibility, making it an attractive alternative to traditional antiseptics. Its incorporation into oral healthcare products such as toothpaste and chewing gum has already demonstrated safety and efficacy.^10^ Coupling LZM with CDs may therefore provide a synergistic strategy to achieve both high antibacterial efficiency and good tissue compatibility.

In this study, we synthesized CDs from biobased precursors (citric acid and urea) via a one-step hydrothermal process and coupled them with LZM. The resulting conjugates (CpCDs) were systematically characterized, and their antibacterial activity and cytocompatibility were evaluated. Such nanocomposites hold promise for a variety of oral applications, including restorative dental materials, implant surface coatings, and adjunctive therapies for periodontal disease, offering a safe and multifunctional approach to infection control in dentistry.

## 2 Materials and Methods

### 2.1 Preparation of Carbon Dots

The CD was synthesized by a solvothermal method. Citric acid (2.88 g, Sigma-Aldrich, Germany) and urea (1.20 g, Sigma-Aldrich, Germany) were weighed and dissolved in 15 mL of a distilled water and acetic acid mixture (v/v is 2:1). The solution was vortexed for 2 min and then transferred to a Teflon-lined autoclave reactor. Hydrothermal treatment was carried out at 200 °C for 12 h. After cooling to room temperature, the resulting product was centrifuged to remove larger particles and sequentially filtered using 0.45 and 0.1 μm PTFE membranes. The clear filtrate was concentrated by using a rotary evaporator, redispersed in 10 mL of distilled water, and filtered again through a 0.1 μm PTFE membrane. Finally, the purified CD solution was dried in a vacuum oven to yield the CD powder.

### 2.2 Preparation of Coupled Carbon Dots

LZM and CD were coupled by using the EDC-NHS method. First, 4 mg of LZM (from hen egg white, Roche Diagnostics GmbH, Germany) was dissolved in 4 mL of Milli-Q water, followed by the addition of 15 mg of 1-ethyl-3-(3′-dimethylaminopropyl) carbodiimide (EDC, Sigma-Aldrich, Burlington, MA) to the solution. The mixture was stirred for 20 min, after which 12 mg of *N*–hydroxysuccinimide (NHS, Sigma-Aldrich, Burlington, MA) was added while the stirring continued for another 30 min to activate the solution. Separately, 60 mg of CD was dissolved in 6 mL of Milli-Q water and then added to the activated solution. The mixture was stirred at 200 r/min for 3 h at room temperature to activate the reaction, followed by an additional 24 h of stirring at the same speed at 37 °C.

Ultrafiltration was used to separate the unreacted reactants from the final products, using ultrafiltration tubes (MWCO = 30,000, Merck Millipore, Burlington, MA) with a relative centrifugal force (RCF) of 1667*g* for 5 min. The supernatant obtained after ultrafiltration was freeze-dried to obtain a purified CpCD powder. The schematic representation of the synthesis protocol is shown in Figure 1.

**Figure 1:**
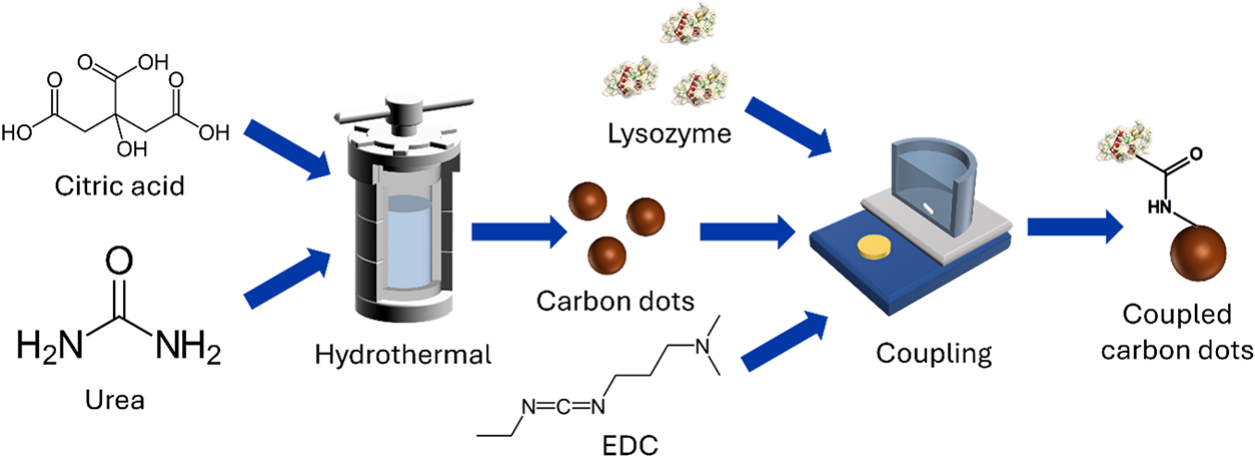
Schematic representation of the study.

### 2.3 Characterization of Carbon Dots, Lysozyme, and Coupled Carbon Dots

#### 2.3.1 Optical and Photoluminescence Property Evaluation

Ultraviolet–Visible (UV–vis) absorption spectra were obtained by using a two-beam scanning spectrometer (Lambda 1050, PerkinElmer). Samples were prepared by dissolving CD, CpCD, and LZM in DI water to a concentration of 0.01 mg/mL.

Photoluminescence (PL) spectra were recorded by using a fluorescence spectrometer (FLS1000, Edinburgh Instruments) equipped with a 450 W ozone-free xenon arc lamp as the excitation source. The CD, CpCD, and LZM samples were prepared at a concentration of 0.1 mg/mL in DI water for these measurements.

#### 2.3.2 Fourier Transform Infrared (FTIR) Spectroscopy Investigation

FTIR spectra of CD, LZM, and CpCD were tested to investigate the functional group change after coupling. FTIR spectra were collected with an FTIR spectrometer (Bruker Vertex 80 vacuum, Bruker Optik GmbH, Ettlingen, Germany). The sticky powder samples were placed on the crystal and detected by a diamond probe in attenuated total reflectance (ATR) mode, as CD and CpCD are viscous pastes.

#### 2.3.3 Zeta (ζ) Potential Measurement

CD, LZM, and CpCD were dispersed in Milli-Q water at a concentration of 5 mg/mL, and then the pH of the solutions was adjusted to 7. The ζ-potentials of CD, LZM, and CpCD were measured at 25 °C by using a Malvern ZetaSizer NanoZS (Malvern, UK). Each sample was measured three times.

#### 2.3.4 Morphology Observation

CD and CpCD were dispersed in Milli-Q water at a concentration of 0.5 mg/mL. The suspensions were dropped on a homemade holey carbon. The morphologies of CD and CpCD were determined with transmission electron microscopy (TEM, Talos F200X, Thermo Fisher Scientific) at an accelerating voltage of 200 kV.

### 2.4 In Vitro Biocompatibility Test

#### 2.4.1 Cell Culture

Human TERT-immortalized gingival fibroblasts (hGFBs, CRL-4061, ATCC) were used for the in vitro biocompatibility test. The cells were cultured in complete culture medium consisting of 10% fetal bovine serum (FBS, Gibco), 1% penicillin/streptomycin, and the balance of Dulbecco’s Modified Eagle Medium (DMEM, Gibco). Cell cultures were maintained in a humidified incubator (MCO-18AIC, Sanyo Electric Biomedical Co., Ltd., Osaka, Japan) with 5% CO_2_ at 37 °C. Cells were passaged when they reached 80–90% confluence in the culture flask and were used for testing after two passages.

#### 2.4.2 Cytotoxicity Test

The suspension (100 μL) containing 50,000 cells/mL was added to each well of a 96-well cell culture plate, and the cells were incubated for 24 h to allow for adherence. CD, LZM, and CpCD were dissolved in complete culture medium and serially diluted 2-fold, starting from 5 mg/mL. The serially diluted solutions were used as test solutions. After 24 h, the culture medium was replaced with serially diluted test solutions, and the cells were incubated for 6, 12, 24, and 48 h. Cell counting kit-8 (10 μL, CCK-8, Sigma-Aldrich, St. Louis, MO) was added to each well, and the samples were incubated for an additional 2 h. Optical density (OD) values for each well were measured using a microplate reader (Multiskan Go, Thermo Fisher Scientific, Vantaa, Finland). Cells cultured in complete medium served as the growth control, while wells containing only complete medium (without cells) were used as the blank control. Each concentration was tested in triplicate.

Cell viability for each test group was calculated using the following equation:

1 $$\mathrm{cell}\;\mathrm{viability}\;\left(\%\right)=\frac{{\mathrm{OD}}_{\mathrm{test}}-{\mathrm{OD}}_{\mathrm{blank}}}{{\mathrm{OD}}_{\mathrm{growth}}-{\mathrm{OD}}_{\mathrm{blank}}}\times 100\%$$

In eq 1, OD_test_, OD_blank_, and OD_growth_ represent the OD values of the test group, blank control, and growth control, respectively.

#### 2.4.3 F-Actin and Nucleus Staining

Round coverslips with a diameter of 12 mm were placed in a 24-well cell culture plate, and hGFBs with a density of 2 × 10^4^ cells/mL were seeded on the coverslips. The cells were then incubated with complete culture medium for 24 h to make them adhere to the coverslips. After the 24 h incubation, the complete culture medium was replaced by the test solutions with concentrations of 5 mg/mL, and the cells were cultured for another 24 h. After the 24 h coculture, the cells were ready to be stained.

The test solution was discarded, and the cells were rinsed twice with phosphate-buffered saline (PBS, 1×, pH 7.4, Gibco) twice. The cells were fixed with 4% paraformaldehyde solution (Merck, Darmstadt, Germany) for 10 min at room temperature and then rinsed twice with PBS. The cells were permeabilized with 0.1% Triton X-100 solution (Merck, Darmstadt, Germany) for 5 min at room temperature and then rinsed with PBS twice. The cells were stained with 200 μL of fluorescein isothiocyanate (FITC) labeled phalloidin (Merck, Darmstadt, Germany) with a concentration of 50 μg/mL for 30 min in the incubator in 5% CO_2_ at 37 °C and were subsequently rinsed with PBS twice. The cells were stained with 200 μL of 4′,6-diamidino-2-phenylindole (DAPI, Sigma-Aldrich, St. Louis, MO) with a concentration of 0.1 μg/mL for 30 s at room temperature and then rinsed with PBS twice. Antifade mounting medium with DAPI (Vector Laboratories, Burlingame, CA) was dropped on glass slides, and the round coverslips were placed with the cells facing down on the glass slides. The fluorescence images of stained cells were captured by an upright fluorescence microscope (Model BX43F, Olympus, Nagano, Japan) in a dark room. During the staining, each rinse took 10 min.

### 2.5 Antibacterial Property Test

#### 2.5.1 Bacterial Culture

*Streptococcus mutans* Ingbritt (*S. mutans* IB) was selected to evaluate the antibacterial properties of the samples in this study. Bacteria were cultured on blood agar plates (Columbia blood agar base, Neogen, NCM 2023, Lansing) supplemented with 5% defibrinated horse blood and incubated in 5% CO_2_ at 37 °C (MCO-17AIC, Sanyo Electric Biomedical Co., Ltd., Osaka, Japan) for 24 h. The bacteria were transferred to another blood plate for passage after 24 h. The bacteria were used for testing after two passages.

#### 2.5.2 Antibacterial Rate Evaluation by the Spread Plate Method

The bacteria were collected from blood agar plates and suspended in different culture media. In this assay, bacteria were cultured with CD, LZM, and CpCD in Mueller–Hinton (M–H) broth (21 g/L, Oxoid) modified in three different ways: complete M–H broth and M–H broth diluted to 20% by addition of normal saline or Milli-Q water, referring to ISO 22196. For convenience, complete M–H broth will hereafter be abbreviated as broth. The broth diluted by normal saline and Milli-Q water will be abbreviated as broth/saline and broth/water, respectively.

The bacterial suspension was adjusted to 10^6^ CFU/mL, and CD, LZM, and CpCD were dissolved with concentrations of 5, 2.5, and 1.25 mg/mL, separately. The bacterial suspension without CD, LZM, and CpCD was set as the growth control. All bacterial suspensions were cultured for 24 h at 37 °C.

After 24 h of culture, the bacterial suspension and growth control were diluted 10^4^ times with M–H broth. The diluted suspension (100 μL) was dropped and was evenly spread on blood agar plates. The plates were cultured in 5% CO_2_ at 37 °C for 24 h. After 24 h of culture, photos were captured, and the colonies on the blood agar plates were counted using micro software ImageJ. The antibacterial rate was calculated according to the equation below.

2 $$antibacterial\;rate\left(\%\right)=\frac{N_{control}-N_{test}}{N_{control}}\times 100\%$$

In eq 2, *N*_control_ and *N*_test_ represent the calculated bacterial densities of the growth control and the test groups, respectively.

#### 2.5.3 Cocultured Bacterial Growth Curve

The *S. mutans* IB bacteria were collected from blood agar plates and suspended in M–H broth, and the OD_600_ was set to 1, representing 10^9^ CFU/mL. The final concentration of bacteria was adjusted to 10^6^ CFU/mL. CD, LZM, and CpCD were dissolved in broth/water with concentrations of 5, 2.5, and 1.25 mg/mL, respectively, as the most distinct difference between different samples was observed in broth/water. The bacterial suspension without CD, LZM, and CpCD was set as growth control. The bacterial density was tested every 6 h by diluting the suspension first and distributing 100 μL of suspension evenly on blood agar plates. The original bacterial density was calculated according to the dilution factor. The antibacterial rate was calculated following eq 2. To meet the requirements of logarithmic coordinate plotting, a minimal offset (10^–1^) was added to the data points with an antibacterial rate of 0.

#### 2.5.4 Experiment of Biofilm Inhibition

The bacteria were collected from blood agar plates and suspended in brain heart infusion (BHI, BD BACTO) broth supplemented with 1% sucrose.^7^ The bacterial suspension was adjusted to 2 × 10^6^ CFU/mL, and 100 μL of the bacterial suspension was seeded into each well of a 96-well cell culture plate. The bacteria were incubated with CD, LZM, and CpCD with the concentration of CD, LZM, and CpCD being 5, 2.5, and 1.25 mg/mL, together. The bacterial suspension without CD, LZM, and CpCD was set as the growth control. The bacteria were incubated anaerobically for 24 h.^7^ The supernatant was discarded after incubation, and then the biofilm was rinsed with sterile water three times. Crystal violet solution (150 μL, 1%, Sigma-Aldrich, Germany) was added into each well, and the biofilm was stained for 15 min at room temperature. The crystal violet solution was discarded, and the biofilm was rinsed at least six times to remove the remaining staining solution that was not attached to the biofilm completely. Ethanol (200 μL, 95%) was added to each well to dissolve bound crystal violet. The dissolving process took 15 min under continuous shaking. The ethanol, with a volume of 100 μL, was then transferred to another 96-well plate, and the OD at 590 nm was measured. The biofilm formation rate was calculated according to the equation below.

3 $$\mathrm{biofilm}\;\mathrm{formation}\left(\%\right)=\frac{{\mathrm{OD}}_{\mathrm{test}}}{{\mathrm{OD}}_{\mathrm{growth}}}\times 100\%$$

In eq 3, OD_test_ and OD_growth_ represent the OD values of the test group and growth control, respectively.

#### 2.5.5 Bacterial Morphology Observation

The bacteria were collected from blood agar plates, and then they were suspended in BHI broth (OD_600_ = 1). The bacterial suspension was diluted to 2 × 10^7^ CFU/mL, and CD, LZM, and CpCD were dissolved with the concentrations of 5, 2.5, and 1.25 mg/mL, separately. The bacterial suspension without CD, LZM, and CpCD was set as the growth control. The bacterial suspension with a volume of 2 mL was incubated in the tubes anaerobically for 24 h since *S. mutans* prefers an anaerobic environment.^7^ The suspension was centrifuged with an RCF of 700*g* for 5 min after incubation. The supernatant was discarded, and the precipitate was rinsed with sterile water three times to eliminate residual CD, LZM, CpCD, and protein in the BHI broth. The bacterial samples were then fixed with 2.5% glutaraldehyde in 0.1 M cacodylate buffer (pH 7.4, Thermo Fisher Scientific) and dehydrated. The bacterial samples were finally dispersed on poly-l-lysine-coated coverslips and observed under a scanning electron microscope (SEM, Carl ZeissTM Evo LS 15, Oberkochen, Germany) at 50 Pa using secondary electron (VPSEE G4) and probe current of 500 pA.

### 2.6 Statistical Analysis

The entire set of experiments was repeated three times. This offset does not affect the statistical analysis of the intergroup differences. The Waller–Duncan’s test, performed using SPSS 29.0, was employed to determine significance, with a threshold of *P* < 0.05 indicating statistically significant differences in the one-way ANOVA. Results are presented as the mean ± standard deviation, and significant differences are marked with an asterisk in the figures.

## 3 Results

### 3.1 Fluorescence Properties

Fluorescence properties of all samples were investigated, including ultraviolet–visible (UV–vis) absorption and photoluminescence (PL) spectra.

In Figure 2a, the normalized UV–vis absorption spectra of both CD and CpCD have an absorption peak at 340 nm and a broad tail over the visible range. LZM presents a main absorption peak at 280 nm,^11^ and a narrow shoulder peak at 290 nm. The spectra of CD and CpCD were similar, demonstrating that, after CD and LZM were coupled, the absorption profile of CpCD predominantly resembled that of CD rather than that of LZM.

**Figure 2:**
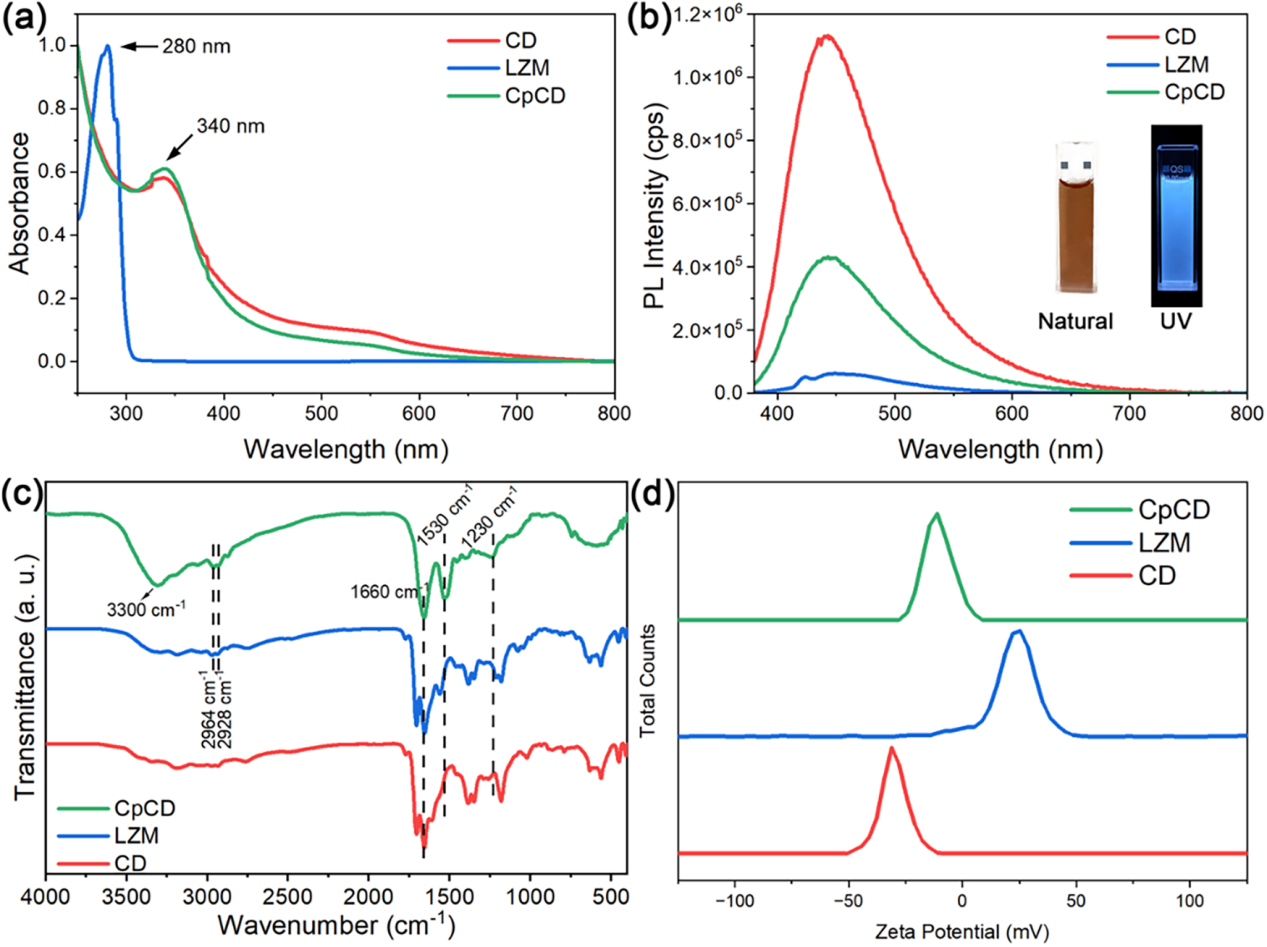
Photoluminescence and physical property characterization of samples: (a) UV–vis spectra, (b) PL spectra, (c) FTIR spectra, and (d) ζ-potentials of CD, LZM, and CpCD.

Figure 2b displays the PL spectra of CD, LZM, and CpCD under ultraviolet excitation at 370 nm. It shows that the maximum emission peaks of CD and CpCD were both at 443 nm in the range of blue light, which is also confirmed by the photo embedded in the figure. The maximum PL intensity of CpCD (4 × 10^5^ cps) was less than that of CD (1 × 10^6^ cps), which was due to the coupled LZM.

The PL spectrum of LZM exhibited two emission peaks at 424 and 448 nm, with intensities of 5 × 10^4^ cps and 6 × 10^4^ cps, respectively. The weak PL intensity indicates that LZM is not a typical photoluminescent material.

### 3.2 FTIR Analysis

FTIR analysis was employed to explore the structural change after coupling by the EDC-NHS method. The normalized FTIR spectra of CDs, LZM, and CpCDs are shown in Figure 2c. Specifically, the spectra of CD and LZM are similar, due to the similar functional groups, such as −CH_3_ symmetric bending (1380–1385 cm^–1^) and −CH_3_ asymmetric bending (1345–1350 cm^–1^) presented in both CDs and LZM. However, the spectrum of CpCD differs from the spectra of CD and LZM, implying that new bonds have been formed during the coupling reaction. It was pointed out that the bands at 2964 and 2928 cm^–1^ in the spectrum of LZM represent the stretching vibration of C–H and absorption of LZM, respectively, and they prove the existence of LZM.^8^ These bands can also be found in the spectrum of CpCD, demonstrating that LZM exists in CpCD.

Furthermore, in the spectrum of CpCD, the band at 3300 cm^–1^ is attributed to N–H stretching vibration in amine groups.^12^ It commonly occurs in the range of 3400–3440 cm^–1^ and is shifted to 3300 cm^–1^ when the amine groups are involved in hydrogen bonds,^13^ and supports the bonding between CD and LZM through the amino group.^8^ The band at 1660 cm^–1^ is assigned to amide I, reflecting the stretching vibration of the peptide carbonyl group (−C=O).^14^ The band at 1530 cm^–1^ in the spectrum of CpCD is assigned to amide II, caused by N–H absorbance,^15^ and the band assigned to amide II can also be found at 1560 cm^–1^ in the spectrum of LZM.^8^ The band at 1230 cm^–1^ in the spectrum of CpCD belongs to the amide III band,^16^ which is associated with N–H bending vibration and C–N stretching.^17^

With these observations in hand, it can be concluded at this stage that LZM was successfully coupled to CD via amide bonds through the EDC-NHS method.

### 3.3 ζ-Potential

ζ-potential, an important parameter in colloid chemistry, also plays an important role in investigating interaction between particles and bacteria.^18^ Figure 2d shows the ζ-potentials of CD, LZM, and CpCD. The ζ-potentials of CD, LZM, and CpCD were −31.0, +25.3, and −11.1 mV, respectively. The ζ-potential of CpCD was between those of CD and LZM, demonstrating that CD and LZM were coupled.

### 3.4 Morphology

The TEM images of CDs and CpCDs are displayed in Figure 3. Severe aggregation has been observed in Figure 3a, and some overlapped CDs are observed at the edge of the cluster in Figure 3b. In contrast, the CpCD dispersed better after coupling (Figure 3c) and the diameter of CpCD was between 12 and 20 nm (Figure 3d). It could be concluded that coupling improved the dispersibility of CD in water.

**Figure 3:**
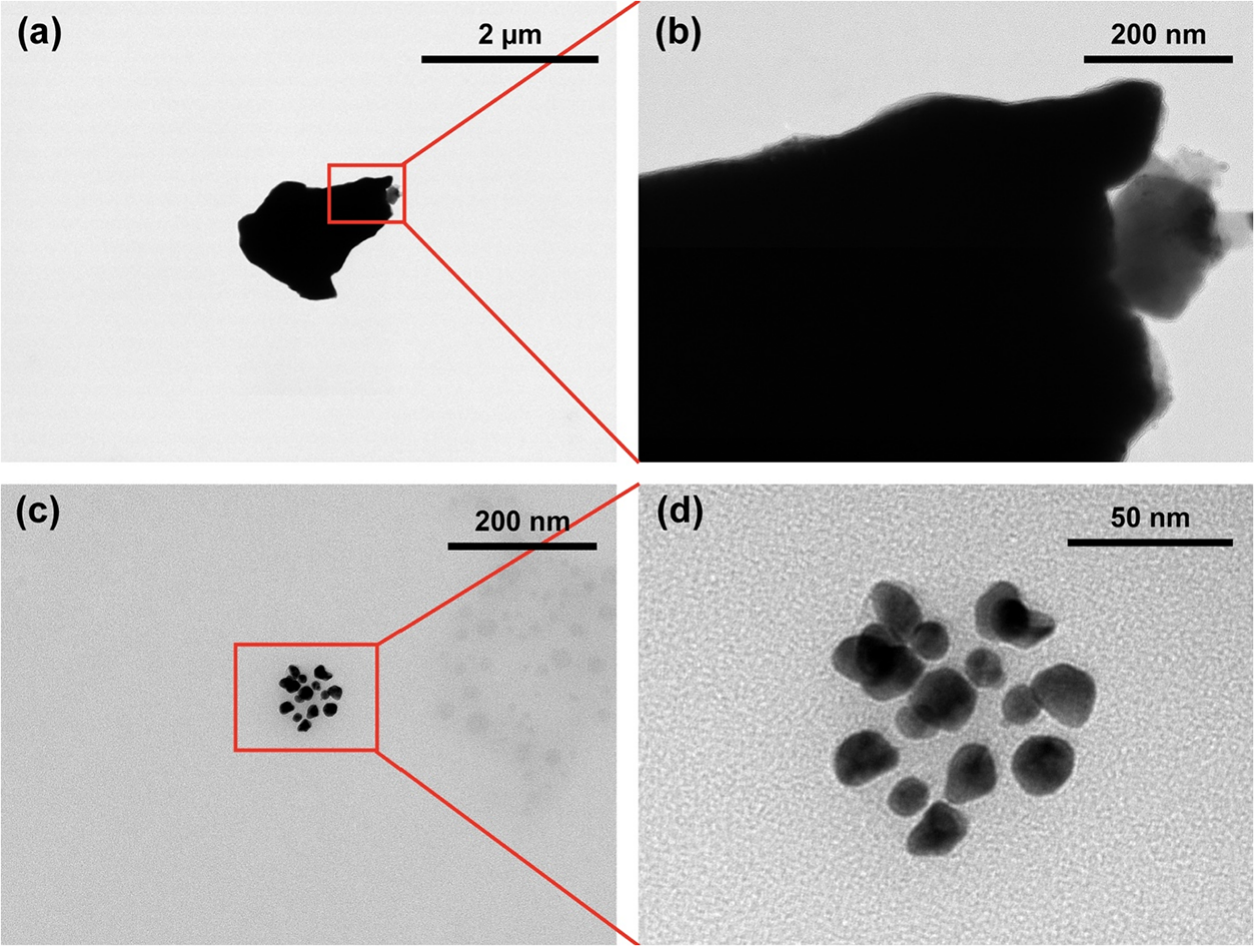
TEM images of (a, b) CD, and (c, d) CpCD at different magnifications.

### 3.5 In Vitro Biocompatibility

The viability of cells cocultured with CD, LZM, and CpCD in 48 h is shown in Figure 4(a–c). In Figure 4a, cell viability increased as time went by when the concentration of CD was lower than 2.5 mg/mL, and CD was regarded as noncytotoxic at the maximum concentration of 2.5 mg/mL within the entire experiment, as cell viability above 75% indicates nontoxicity.^19^ In contrast, it decreased with a concentration of 5 mg/mL, and it showed cytotoxicity after 12 h coculture. As for LZM, it showed no cytotoxicity in the range of 5 mg/mL to 9.77 μg/mL. The viability of cells cocultured with LZM was close to that of the control group as it fluctuated around 100%. Figure 4c shows that CpCD was not cytotoxic to cells in the range of 5 to 9.77 μg/mL, implying that coupling could enhance the biocompatibility of CD.

**Figure 4:**
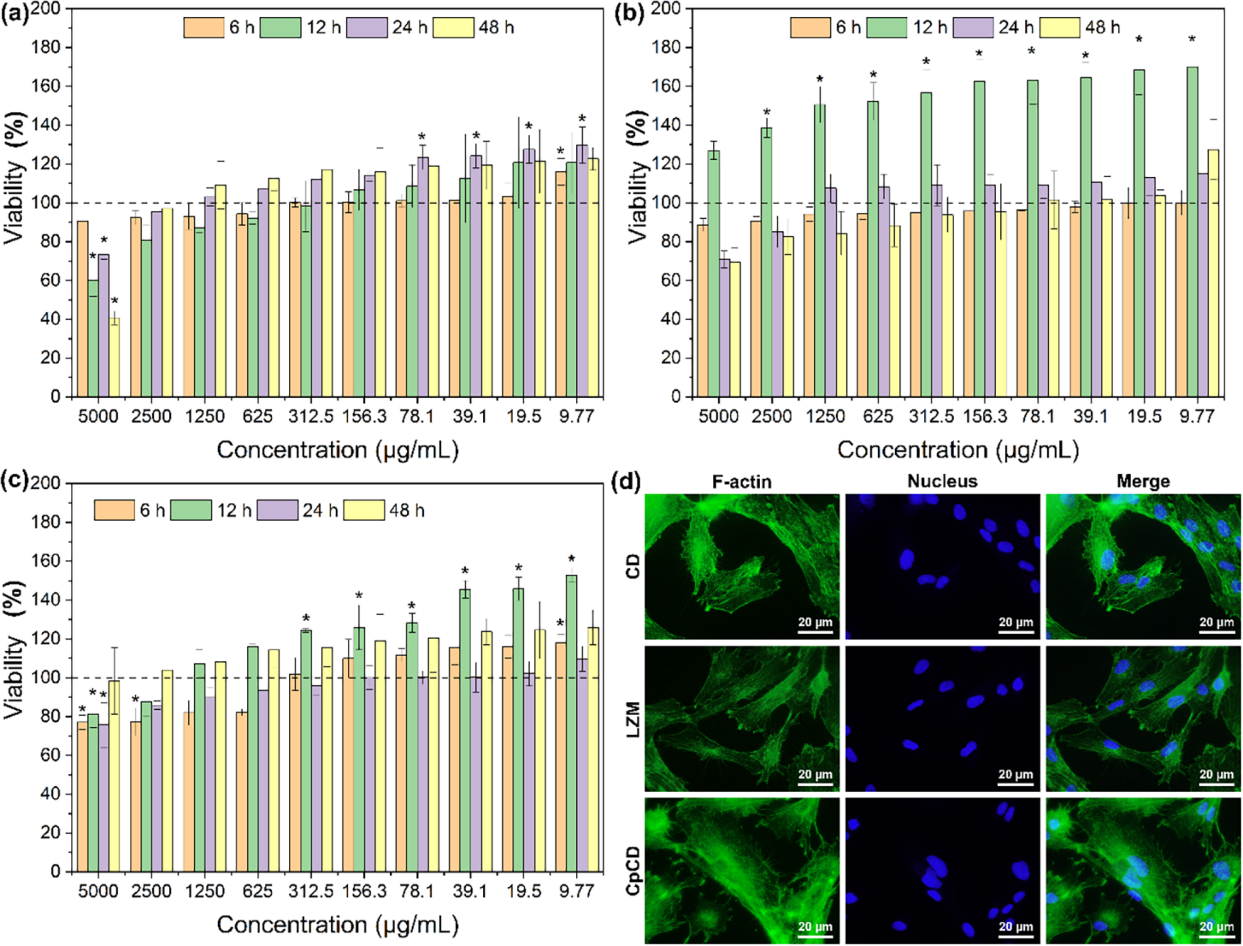
Viability of cells cocultured with (a) CD, (b) LZM, and (c) CpCD for 48 h. (d) Fluorescence images of F-actin and nuclei cocultured with CD, LZM, and CpCD for 24 h.

The influence of CD, LZM, and CpCD on cells was investigated by observing the cell morphology. The nuclei and F-actin were stained, and they emitted blue and green fluorescence under a fluorescence microscope. F-actin is widely present in the cytoskeleton; therefore, the morphology of the cells could be shown.

In Figure 4d, cells were observed in a good state incubated with 5 mg/mL CD, LZM, and CpCD. The cells were stretched, and the nucleus accounted for a small proportion of the cells. Moreover, filopodia were also observed cocultured with all samples. Regarding the morphology of cells, there was no obvious difference between cells cocultured with CD, LZM, and CpCD, and therefore, it can be concluded that cells could grow and proliferate in the presence of CD, LZM, and CpCD with a maximum limit of 5 mg/mL.

### 3.6 Antibacterial Property

The antibacterial property of CD, LZM, and CpCD was characterized by the spread plate method. Considering the balance of the bacterial growth rate and inhibitory effect of materials, three media were employed in the experiment. Two diluted M–H broths were used to slow bacterial growth, referring to ISO 22196. The bacteria were cocultured with different materials for 24 h, and then the bacterial density was calculated by counting the colony number on the blood plates, which is shown in Figure 5a–c. The antibacterial rate was also calculated based on the bacterial density and is shown in Figure 5d–f.

**Figure 5:**
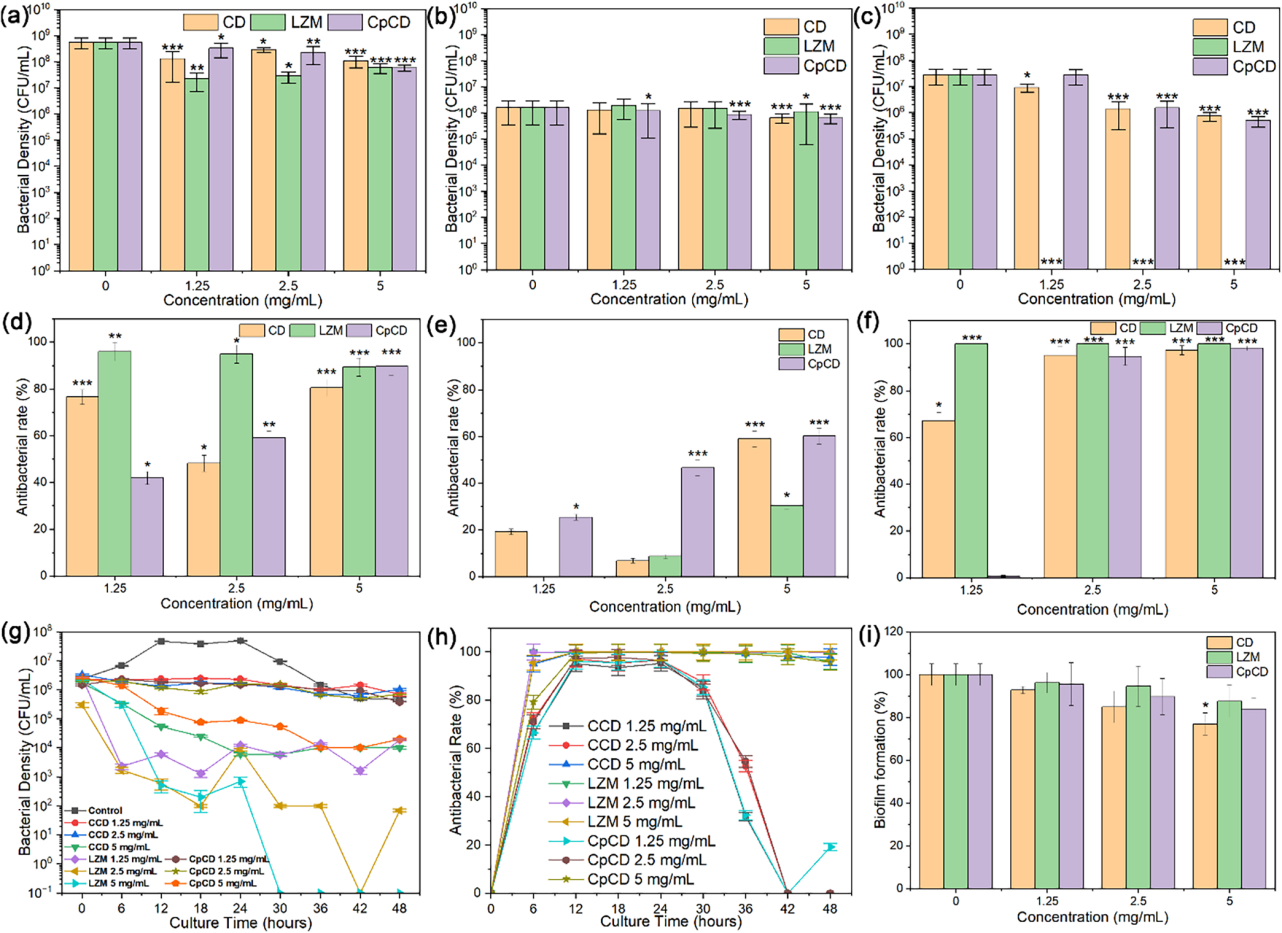
Density of bacteria cocultured with CD, LZM, and CpCD in (a) broth, (b) broth/saline, and (c) broth/water for 24 h. Antibacterial rate of CD, LZM, and CpCD in (d) broth, (e) broth/saline, and (f) broth/water for 24 h. Changes in (g) bacterial density and (h) calculated antibacterial rate during coculture with CD, LZM, and CpCD in broth/water for 48 h. (i) Biofilm formation rate of bacteria incubated with CD, LZM, and CpCD for 24 h.

In Figure 5a, the bacterial density reduced by 1 order of magnitude in the concentration range of 1.25 and 5 mg/mL in broth, confirming the inhibitory effect of LZM on *S. mutans*. It is consistent with the reported result revealing that LZM can effectively inhibit Gram-positive bacteria, including *S. mutans*.^8^ As for CD, although bacterial density decreased, the decrease was not significant. Similarly, at the concentrations 1.25 and 2.5 mg/mL, the decrease was not significant for CpCD either. However, when the concentration reached 5 mg/mL, the bacterial density was reduced to the same level as that obtained when cocultured with LZM, which may benefit from LZM coupled with CD. The inhibitory effect of LZM and CpCD at 5 mg/mL is more clearly displayed in Figure 5d, where the antibacterial rate of LZM in the range of 1.25 and 5 mg/mL, and CpCD at 5 mg/mL was higher than 90%.

In Figure 5b,c, the bacterial density of the control group was less than that in Figure 5a, demonstrating that the bacterial growth was delayed. Among them, bacteria grew slowly in broth/water, while bacterial density remained at the same level as the initial level (10^6^ CFU/mL), reaching a dynamic balance. In Figure 5b, the bacterial density reduced slightly for all test groups, including LZM, and they showed no obvious inhibitory effect in Figure 5e, spontaneously.

Inhibited bacterial growth was more obvious in broth/water, as shown in Figure 5c,f. LZM killed almost all of the bacteria in broth/water and thus displayed an antibacterial rate of 100%. The bacterial density was negatively correlated with material concentration after being cocultured with CD and LZM for 24 h. The bacterial density reduced by 1 order of magnitude when the concentration of CD and CpCD reached 2.5 mg/mL in Figure 5c, and their antibacterial rate was more than 90% in Figure 5f.

The dynamic change in the bacterial density in broth/water is shown in Figure 5g. As the curve of the control group shows, bacteria grew rapidly in the first 12 h, and after that, they maintained a stable density of 4 × 10^8^ CFU/mL from 12 to 24 h. The bacterial density reduced after 24 h and then remained at the same level as the initial state after 42 h. Regarding CD and CpCD at low density (1.25 and 2.5 mg/mL), the bacterial growth was inhibited as there was no such stage contributing to the bacterial growth from 12 to 24 h. In contrast, the bacterial density decreased during coculturing with CD and CpCD at 5 mg/mL. The bacterial density ultimately decreased by 2 orders of magnitude at this concentration. As for LZM, it showed a significantly stronger inhibitory effect than CD and CpCD. When concentration of LZM was 1.25 mg/mL, the growth curve was close to those of CD and CpCD with a concentration of 5 mg/mL, but the bacterial density reduced more rapidly in the beginning. When concentration of LZM was over 2.5 mg/mL, it showed a continued inhibitory effect on bacteria, and the bacterial density decreased to zero at the end of the test, confirming the excellent antibacterial property of LZM.

Change of antibacterial rate, which was calculated based on the bacterial density, demonstrates the percentage of bacterial proliferation inhibited more obviously (Figure 5h). All test groups were divided into three groups. The first group includes CD with a concentration of 5 mg/mL and LZM with a concentration between 1.25 and 5 mg/mL. The antibacterial rate of the first group increased to almost 100% rapidly after a 6 h coculture, and the high antibacterial rate was maintained until the end of the test after 48 h, demonstrating that this group has both excellent and stable antibacterial effect. The second group includes CD and CpCD with a concentration lower than 2.5 mg/mL, whose antibacterial rate reached almost 100% after a 12-h coculture but declined after the 24 h coculture. After 36 h, the antibacterial rate was only about 50%, and after 42 h, it dropped to 0. The results indicate that when the concentrations of CD and CpCD are below 2.5 mg/mL, they can inhibit bacterial growth effectively for up to 12 h. The third group only includes CpCD with a concentration of 5 mg/mL. The antibacterial rate trend of the third group was between those of the first group and the second group. The antibacterial rate of the third group was around 80%, close to that of the second group at 6 h. However, the antibacterial rate of the third group did not decrease and remained close to 100% after 24 h, as in the first group, implying that although the antibacterial effect worked slowly initially, CpCD also had a stable antibacterial effect during the whole test.

Biofilms can provide long-term protection for microorganisms,^20^ and thus biofilm inhibition investigation is also necessary. The biofilm formation after 24 h is displayed in Figure 5i. Biofilm inhibition was negatively correlated to the concentration of materials. Furthermore, CD inhibited biofilm formation the most when the concentrations of CD, LZM, and CpCD were the same. When the concentration of CD, LZM, and CpCD was at 5 mg/mL, they inhibited about 23, 12, and 16% of biofilm formation, respectively.

Figure 6 shows the SEM images of bacteria cocultured with samples. *S. mutans* are spherical cells that connect to form chains. Most bacterial cells were embedded in a porous fibrous structure, which is speculated to be a biofilm.^21^ Compared with the control group, some particles were found on the bacteria cocultured with CD, LZM, and CpCD, which are shown in Figure 6b–e. These particles are speculated to be related to the interaction between the sample and the bacteria. In Figure 6c, most bacteria were observed only inside the biofilm, implying that most bacteria on the surface were hydrolyzed by LZM and only the bacteria inside survived. This phenomenon is consistent with previous results, showing that LZM effectively inhibits *S. mutans*. Furthermore, hydrolyzed bacterium was also captured in the LZM group, which is marked in the red circle in Figure 6d. As for the CpCD group, some deformed bacteria were also observed, which are marked in the red circle in Figure 6e. It shows that CpCD had an improved inhibition effect on *S. mutans* compared with CD.

**Figure 6:**
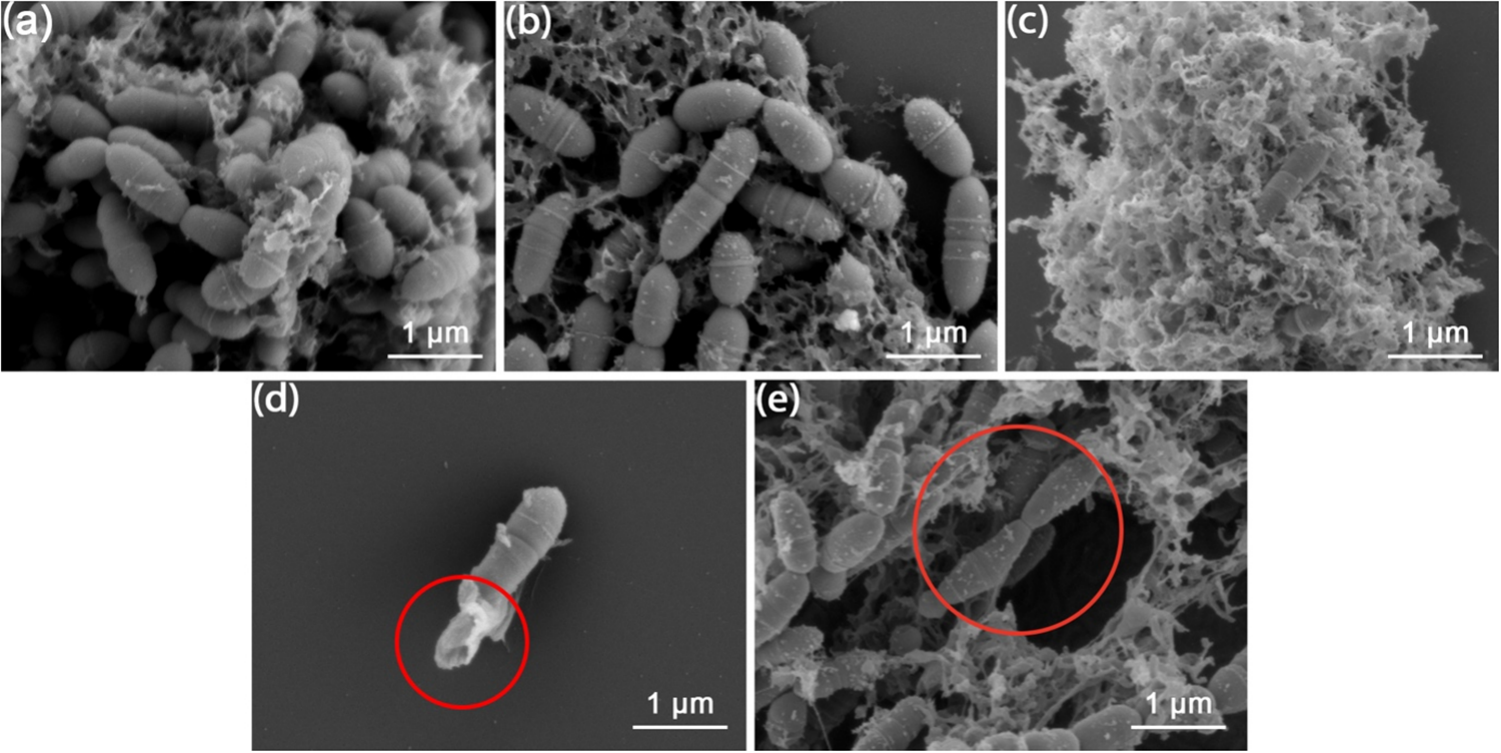
SEM images of bacteria cocultured (a) without samples (control), (b) with CD, (c, d) LZM, and (e) CpCD.

## 4 Discussion

In this study, the coupling of CDs and LZM has been realized by means of covalent amide bond by using EDC and NHS, which is a common coupling method in biochemical conjugations, especially in peptide synthesis.^22^ Lin et al. summarized that there are 11 free carboxyl groups on the surface of LZM in total based on studies of Phillips et al. When LZM was mixed with EDC, the active O-acylisourea intermediate formed by EDC reacting with carboxyl groups.^23^ However, O-acylisourea is unstable. Thus, NHS is usually introduced to stabilize O-acylisourea by converting O-acylisourea to a more stable NHS ester. During hydrothermal synthesis of CD, urea was added to introduce amino groups to CD. The amino group formed an amide group with the original carboxyl group, which was confirmed by FTIR results (Figure 2c), and the ζ-potential of CpCD is between that of CD and LZM (Figure 2d), which also confirms that CD and LZM were coupled by the EDC-NHS method.^8^ EDC byproduct was released as a soluble urea.

Considering the molecular size of LZM, which is between 3 and 5 nm, is smaller than that of CD, we invested an excessive amount of LZM to make CD fully react. We estimated the required amount of LZM to control excessive CD during the reaction. Based on the estimated molecular weight of CD (3500 g/mol) and a nitrogen content of 14.6 atom %, 60 mg of CD corresponds to approximately 2.5 × 10^–6^ mol of amino groups.^24^ To ensure complete reaction of CD, the amount of LZM should therefore provide at least 2.5 × 10^–6^ mol of carboxyl groups, which corresponds to a minimum of 3.3 mg of LZM as the molecular weight of LZM is 14,400 g/mol. Accordingly, an excess amount of LZM (4 mg) was selected to ensure that CD reacted as completely as possible. In this way, residual LZM could be discarded with EDC, NHS, and EDC byproducts together during ultrafiltration.

Biocompatibility is a prerequisite in biomaterial study, as a qualified biomaterial should be safe and harmless. Considering the amount of lysozyme in tears could be upper to 5 mg/mL,^25^ we also investigated the in vitro biocompatibility and antibacterial effect of CD, LZM, and CpCD with a concentration lower than 5 mg/mL. Combining cytotoxicity test results and cell staining results, LZM and CpCD were regarded nontoxic when their concentration was up to 5 mg/mL, which is consistent with the reported results. Although the in vitro biocompatibility of CD was not as excellent as LZM and CpCD, it was considered nontoxic with a concentration up to 2.5 mg/mL. CD and CpCD showed an acceptable biocompatibility with a relatively high concentration compared with reported studies in copper-doped carbon dots, citric acid CDs,^7^ and LZM-bonded 4-aminosalicylic acid CDs,^8^ where biocompatible concentration is 240 μg/mL, which means that CpCD has a more flexible application concentration.

Dental caries is a common chronic infectious disease resulting from tooth-adherent cariogenic bacteria, primarily *S. mutans*, a Gram-positive bacterium that produces acid contributing to tooth demineralization. There are two main ways to inhibit dental caries, including remineralization and bacterial inhibition. Fluoride is currently the most common and effective substance, which can replace hydroxyl groups to form more erosion-resistant minerals.^26^ As for bacterial inhibition, chlorhexidine, alcohol, etc., are the most common ingredients in commercial products, as mentioned in the Introduction, where their advantages and shortcomings have been discussed.

In this study, LZM was coupled with CD with the expectation of improving the antibacterial properties of CD. CpCD displayed a desirable result: an antibacterial rate of 95% at 2.5 mg/mL (Figure 5f), which was within the biocompatible range. Moreover, CpCD could exert its antibacterial effect continuously for at least 36 h, as compared to CD, which inhibited bacterial growth for only 12 h with the biocompatible range (2.5 mg/mL), as shown in Figure 5h. CpCD also inhibited biofilm formation more than LZM did (Figure 5i). At the same time, the antibacterial duration and biofilm inhibition effect of CpCD exceeded those of the reported lysozyme cross-linked carbon dots,^8^ whose antibacterial duration are between 12 and 24 h. Furthermore, coupling LZM and CD also improved the dispersibility of CpCD in water, which makes it more suitable in alcohol-free mouthwashes. The photoluminescence property of CpCD may make it promising for fluorescence imaging.

ζ-potential characterizes the surface charge of particles. It is worth mentioning that LZM had a positive ζ-potential, whereas the ζ-potentials of CD and CpCD were negative. Olsson et al. measured ten different strains of *S. mutans*, and the results displayed that all tested strains had a negative ζ-potential in normal medium when pH was higher than 3.^27^ This charge disparity may facilitate the coupling between CD and LZM; however, it also may hinder the attachment of CpCD to the bacterial surface, potentially weakening its antibacterial effect. Meanwhile, some negatively charged carbon dots were reported with excellent antibacterial properties. Kung et al. synthesized negatively charged carbon dots from citric acid and urea showing antibacterial properties; however, the mechanism was not explained.^28^ Nocito et al. prepared negatively charged antibacterial carbon dots from Mediterranean olive solid waste and found that carbon dots with strong positive or negative charges both exhibit enhanced antibacterial activity.^29^ It can be inferred that the antibacterial property should decrease when negatively charged carbon dots are coupled with positively charged lysozyme. However, CpCD with an intermediate charge still exhibited excellent antibacterial properties, which may be due to the synergistic effect of the antibacterial properties of carbon dots themselves and the hydrolysis of lysozyme.

Above all, while CDs have been widely explored in various biomaterial applications, their use in dental materials remains underrepresented.^30^ A novel nano dental material was synthesized in our study and displayed desirable antibacterial properties. The synthesis method provided a feasible scheme for bonding CD with natural enzymes, and the antibacterial investigation also provided experience on experiments of nanomaterials on dental pathogens.

## 5 Conclusions

In this study, CDs were synthesized from citric acid and urea by a solvothermal method and coupled with LZM via a one-step EDC-NHS reaction. The amide linkage improved dispersibility, and CpCD showed particle sizes of 12–20 nm in TEM images. CDs were biocompatible up to 2.5 mg/mL, whereas CpCD increased this threshold to 5 mg/mL, confirming the enhanced biocompatibility. Moreover, the antibacterial activity of CpCD was superior, extending the inhibition of *S. mutans* from 12 to 36 h. These results suggest CpCD as a safe, efficient, and water-soluble antibacterial nanomaterial in future applications in oral healthcare and medicine.
